# Knowledge, attitude, and determinants of exclusive breastfeeding during COVID-19 pandemic among lactating mothers in Mekelle, Tigrai: a cross sectional study

**DOI:** 10.1186/s12884-022-05186-w

**Published:** 2022-11-18

**Authors:** Gebretsadkan Gebremedhin Gebretsadik, Zuriash Tadesse, Liya Mamo, Amaha Kahsay Adhanu, Afework Mulugeta

**Affiliations:** 1 Department of Nutrition and Dietetics, School of Public Health, College of Health Sciences, Mekelle University, Mekelle, Ethiopia; 2 Department of Biostatistics, School of Public Health, College of Health Sciences, Mekelle University, Mekelle, Ethiopia

**Keywords:** EBF, COVID-19, Tigrai, KAP

## Abstract

**Background:**

Due to the nutritive and immunologic benefits of breastmilk, children should be exclusively breastfed for the first 6 months of life, even during the corona virus pandemic. However, fear of transmission risk and pandemic-related restrictions could undermine the practice of breastfeeding. This study aimed to assess the knowledge, attitude, and determinants of exclusive breastfeeding (EBF) during COVID-19 among lactating mothers in Mekelle, Tigrai, Ethiopia.

**Methods:**

A community based cross-sectional study was conducted among 621 lactating mothers living in Mekelle city, Tigrai, from April to June, 2021. Data were collected using an adapted form of a standard KAP questionnaire. Binary logistic regression was used to determine the independent determinants of EBF at a statistical significance of *p* < 0.05. The strength of the association was measured by odds ratio and 95% confidence interval.

**Results:**

Four hundred (64.4%) mothers exclusively breastfed their children. Infants from female-headed households had twice (AOR 2.21; 95% CI 1.31, 3.71) higher odds of EBF. Higher educational status was associated with higher odds of EBF practice. A unit increase in parity was associated with a 23% increase in the odds of EBF. Mothers who received breastfeeding information had a 73% (AOR 1.73; 95% CI 1.17, 2.56) higher odds of EBF. Moreover, mothers with high knowledge score and positive attitude showed a 74% higher (AOR 1.74; 95% CI 1.20, 2.51) and more than double (AOR 2.35; 95% CI 1.50, 3.70) odds of EBF, respectively.

**Conclusion:**

About two-thirds of the mothers practiced EBF. Household head, maternal educational, parity, breastfeeding information, knowledge of breastfeeding, and attitude towards EBF were significant determinants of EBF. Our study findings highlighted that programs that enhance women’s participation in education and decision-making could improve EBF practice. Besides, during the COVID-19 pandemic, providing lactating mothers with adequate and up-to-date breastfeeding information could be significant in improving EBF practice.

## Background

Breastmilk is the first natural food for babies that solely provides adequate nutrition for infants, especially in the first 6 months of life [1]. Breastfeeding protects infants and children from infectious diseases by strengthening the immune system via direct transfer of antibodies from the mother [2]. Due to such protective benefits, the World Health Organization (WHO) recommends exclusive breastfeeding for the first 6 months of life, initiation of breastfeeding within an hour of birth, and continued breastfeeding up to the age of 2 years and beyond, along with proper complementary feeding, as optimal breastfeeding practices [3, 4]. Exclusive breastfeeding (EBF) is defined as consumption of only breast milk by an infant from a birth mother, wet nurse, or expressed breastmilk with addition of no other liquid or solid except drops or syrups consisting of vitamins, minerals supplements, or medicine for the first 6 months [3].

Corona Virus Disease-19 (COVID-19), a disease caused by severe acute respiratory syndrome coronavirus 2 (SARS-CoV-2), was first identified in Wuhan, China and declared a pandemic after creating a world health crisis [5]. The virus is capable of rapid person to person spread through respiratory droplets that approach the mucous membranes of the mouth, nose, or eyes during coughing, sneezing, talking, or through direct hand contact with contaminated objects [5, 6].

The WHO recommends mothers with suspected or confirmed COVID-19 to initiate or continue breastfeeding and emphasizes that the benefits of breastfeeding significantly outweigh the possible risks for transmission [7]. For a mother who tested positive for COVID-19, it’s more beneficial if she begins to or keeps breastfeeding her child through skin to skin contact following the necessary precautions including wearing a face mask, washing hands before and after contact with her child, and routinely cleaning any surfaces she touches [7–9]. Nonetheless, separation of the infant from the mother might lead to a greater risk of infection with other serious pathogens in the child that would be prevented through EBF [9, 10].

In Ethiopia, only 59% of mothers/caregivers practice EBF [11]. In addition, the magnitude of infant and under-five mortality is 43 and 55 out of 1000 live births, respectively [11]. The part that optimal breastfeeding practices play in preventing infant and under-five deaths in developing countries has already been established [12]. Hence, in the recently endorsed food and nutrition policy of Ethiopia, promoting optimal breastfeeding practices is regarded as one of the main nutrition specific strategies to fight malnutrition [13].

Before the COVID-19 pandemic, several studies have assessed the Knowledge, Attitude, and Practice (KAP) of lactating mothers towards EBF. Maternal knowledge and attitude, maternal level of education, occupation, maternal health, and age of the child were some of the factors found to significantly influence the practice of EBF [14, 15]. These had led to the development, recommendation, and application of local education programs primarily focusing on infant and child feeding and nutrition [16].

Due to fear of risk of transmission, limited knowledge, undesirable attitude, or inappropriate practice during the COVID-19 pandemic, significant number of lactating mothers may stop or decrease to exclusively breastfeed their infants, which may backfire with more serious consequences. Besides, several post-pandemic restrictions to the health service system may have prevented mothers from getting adequate, proper, and up to date breastfeeding-related information. Despite the fact that similar studies conducted before the pandemic exist, none has studied the KAP of lactating mothers on EBF and its determinants during the COVID-19 pandemic. This study could help local health care providers and programmers in devising informed, tailored, and evidence based interventions. It could also be used as a baseline input for experimental studies working on breastfeeding and COVID-19 risk of transmission. Therefore, the aim of this study was to assess the KAP of EBF during COVID-19 and its determinants among lactating mothers in Mekelle, Tigrai, Ethiopia.

## Methods

### Study design and setting

A community based cross-sectional study was conducted among lactating mothers living in Mekelle city, Tigrai, from April to June 2021. Mekelle, the capital city of Tigrai, is located around 780 km north of the Ethiopian capital Addis Ababa, with an elevation of 2254 m above sea level. Administratively, it is considered a special Zone which is divided into seven sub-cities namely Hawelti, Adi-Haki, Kedamay Weyane, Hadnet, Ayder, Semien and Quiha. It has one comprehensive specialized hospital, three general hospitals, nine health centers, and several private clinics. Mekelle was selected for this study because it represented the highest number of COVID-19 cases in Tigray [17].

### Study participants

The source population for this study were all lactating mothers and/or care givers with infants aged under 6 months that lived in Mekelle city during the study period. Inclusion criteria: 1) Being a lactating mother with an infant under the age of 6 months; 2) Permanent residence in Mekelle city. Exclusion criteria: 1) Unwillingness to participate; 2) Having a psychiatric illness.

### Sample size determination

The sample size for this study was determined using the single proportion formula for cross-sectional studies as follows:

$$n=\frac{Z^2 pq}{d^2}$$

Where, *n* = the required sample size, *Z* = the standard normal deviation at 95% confidence level (1.96), *p* = the estimated proportion of the mothers who practiced EBF (50%), *q* = 1-*p*, *d* = margin of error (0.05), design effect of 10%, and a 10% non-response rate. This yielded a total sample size of 633.

### Sampling procedures

First, four sub-cities (Adi-Haki, Kedamay Weyane, Ayder, and Hawelti) were selected from the total seven by lottery method. Second, two “kebelles” from each sub-city were randomly selected. Kebelle is the local name for the lowest administration unit. Next, the number of respondents to be included in the sample from each kebelle was determined proportionally to the total number of lactating mothers in each of the selected kebelles. This was done by dividing the number of households with mothers/care giver-infant dyads in a specific kebelle by the total number of households with mothers/care giver-infant dyads in all kebelles and multiplying it by the total sample size. Households to be included in the study were traced from a sampling frame that was prepared with the help of local community health centers found in each of the selected kebelles.

### Data collection tools and procedures

Data were collected through an interviewer administered structured questionnaire. The questionnaire solicited data on: 1) Demographic and socioeconomic characteristics that included child age and sex, maternal age, educational status, occupation, religion, monthly income, etc.; 2) Health service and obstetric-related factors including parity, antenatal care, type of delivery, place of delivery, breastfeeding information, COVID-19 status, etc.; 3) Knowledge questions; 4) Attitude questions, and 5) Practice related questions.

### Knowledge, attitude, practice (KAP) tool and variables

The KAP of the mothers towards EBF during COVID-19 was assessed by a KAP questionnaire adapted from the Food and Agriculture Organization of the United Nations (FAO) guidelines for assessing nutrition-related knowledge, attitudes, and practices (KAP) manual. The FAO manual proffers a direction to effectively carry out and analyze community level nutrition-related KAP surveys. It contains validated and reliable modules that gather information on critical KAP related to the 13 most common nutrition issues [18]. It also is helpful in the standardization of KAP studies and thus in comparing their results. The questionnaire, particularly the one about feeding infants 0-6 months (Module 1), was adapted in a way that took into account the objectives of this study and the recent WHO recommendation on breastfeeding during COVID-19.

Maternal knowledge of breastfeeding during COVID-19 was assessed using 16 questions and a score was generated for each mother based on the number of correctly answered questions. This was done by according one point for each correct response and no point for each wrong response. Knowledge was then categorized into high (those who correctly answered > 70% of the questions) and low (those who correctly answered 70% or below of the questions) categories [18].

Maternal attitude towards breastfeeding during COVID-19 was measured by 6 questions. The questions assessed mothers’ perceived benefits and barriers to exclusively breastfeed their children and breastfeeding on demand as well as self-confidence in expressing and storing breastmilk during the COVID-19 pandemic. The questions were measured on a 3-point Likert scale of a positive response, a middle option for uncertain attitudes, and a negative option. Finally, the attitude variable was grouped into high (those who correctly answered > 70% of the questions) and low (those who correctly answered 70% or below of the questions) categories [18].

Questions used to assess practice of EBF included but were not limited to recall of EBF in the last 24 hours, breastfeeding by cup, spoon, or wet nurse in the previous day, breastfeeding status in the mother’s absence, and introduction of liquids (i.e. plain water, infant formula, tinned milk, powdered or fresh animal milk, juice/juice drinks, clear broth, yogurt, porridge, herbal teas, solid/marshy foods) in the previous 24 hours. More specifically, a mother was said to be practicing EBF if she exclusively breastfed her baby in the previous day with no other food or drink, not even water. Breastfeeding by a wet nurse, feeding of expressed breast milk, consuming prescribed medicines, oral rehydration solution, or vitamins and minerals were also regarded as EBF. However, herbal fluids and similar traditional medicines are counted as fluids, and hence consuming such items was not considered as EBF.

### Data quality control

Three data collectors, who were native Tigrigna language speakers and who had similar previous experiences, with a minimum of a Diploma in Health related discipline were recruited. The data collectors were trained for 3 days on study objectives, data collection techniques, recording of responses, research ethics, and COVID-19 prevention precautions.

The tool was reviewed by a team of nutritionists and dietitians in terms of its content and appropriateness to allow for the inclusion of local foods commonly given to infants in the study area. It was also translated into Tigrigna, the local language, and then back translated to English to check for consistency and accuracy. Finally, the Tigrigna language version of questionnaire was used for data collection. Before the actual data collection commenced, the questionnaire was pilot tested on 10% of the sample that created room for any necessary amendments and modifications. Besides, it was manually checked for completeness and consistency. Finally, an amended and corrected form of the Tigrigna version of the questionnaire was used for data collection.

The data collection process went under strict precautions of COVID-19 prevention. Both the interviewer and the interviewee wore face masks and maintained at least two meters of physical distance between them. They also thoroughly washed their hands using a soap or alcohol based hand sanitizer.

### Data analysis and presentation

After checking for completeness and consistency, data were entered into EPI-INFO 3.5.1 Software before being exported to STATA SE 16.0 for statistical analysis. Descriptive statistics for categorical variables are presented using frequencies and percentages. Continuous variables are presented by means and standard deviation or/and median and interquartile range (IQR). Cross tabulation and chi-square tests were used to show univariate associations.

Binary logistic regression was run in two steps to determine independent predictors of EBF practice during COVID-19 pandemic. The first step involved a bivariate logistic regression of each possible independent variable against the dependent variable. In the second step, variables that were associated with the dependent variable at *p* ≤ 0.20 in the first step were entered into a multiple logistic regression model. Finally, only those variables statistically significant at *p* < 0.05 were kept in the final model. Model fitness was tested by Hosmer and Lemeshow goodness-of-fit. Multi-collinearity among independent variables was also assessed using variance inflation factor using the value of 10 as a cut off. Results are presented using odds ratios and their respective confidence intervals at 95%.

## Results

### Socio-demographic and economic characteristics

Out of 633 sampled respondents, 621 completed the questionnaire yielding a response rate of 98.1%.

Five hundred (80.5%) households were male-headed. The mean (±SD) age of mothers/caregivers was 27.6 (4.9) years. The mean (±SD) and median (IQR) family size were 4.6 (±1.5) and 4 (2), respectively. Additionally, all respondents were urban dwellers and majority (87.8%) were Orthodox Christians in religion. Regarding educational status of the mothers, majority (97.3%) attended formal education. Moreover, 401 (64.5%) and 220 (35.5%) mothers/caregivers were homemakers and employed, respectively. The mean (±SD) and median (IQR) infant age were 2.8 (±1.39) and 3 (2) months, respectively. Furthermore, more than half (58.6%) of the infants were females in sex (Table 1).

**Table 1:** Socio-economic and demographic characteristics of mothers or caretakers of infants in Mekelle city, Tigrai, 2021 (*n* = 621)

Variable (Missing)	Category	Frequency (%)
Household head sex(0)	Male	500 (80.5)
	Female	121 (19.5)
Religion (0)	Orthodox	545 (87.8)
	Muslim	76 (12.2)
Marital Status (0)	Currently unmarried^a^	58 (9.3)
	Currently married	563 (90.7)
Mother’s education (0)	No formal education	17 (2.7)
	Formal education	604 (97.3)
Mother’s occupation (0)	Housewife	401 (64.5)
	Employed	220 (35.5)
Monthly household income in ETB (0)	Mean (SD)	8164.6 (5598.9)
	Median (IQR)	7000 (5800)
Infant sex (0)	Male	257(41.4)
	Female	364 (58.6)

*ETB* Ethiopian birr

^a^Single, divorced, or widowed

### Health and obstetric characteristics

Majority (96.9%) of the mothers/caregivers had at least one antenatal care visit for their last pregnancy. Similarly, of the 602 mothers who reported to have made antenatal visits, 590 (98%) were attended by skilled health professionals. The mean (±SD) parity stood at 2.58 (±1.51) births. Out of five hundred seventy seven (92.9%) deliveries at health facilities, 158 (25.4) were by caesarean section (CS). Additionally, nearly three-fourth (71.8%) of the mothers reported to have received breastfeeding information post-delivery. Moreover, only 7 (1.1%) and 9 (1.5%) mothers/caregivers reported to be ever infected with COVID-19 and jabbed with COVID-19 vaccine, respectively.

### Knowledge of exclusive breastfeeding during COVID-19

Almost all mothers (99.5%) knew that the first food for the newborn is breastmilk. Similarly, about 610 (98.2%) mothers correctly defined EBF. Besides, 401 (64.6%) mothers did not know that a COVID-19 positive/suspect lactating mother should initiate/continue breastfeeding with strict precautions (Table 2).

**Table 2:** Knowledge of breastfeeding during COVID-19 among lactating mothers/caregivers in Mekelle city, Tigrai, 2021, (*n* = 621)

Variable	Category	Frequency (%)
First food for the newborn is breastmilk	Knows	618 (99.5)
	Doesn’t know	3 (0.5)
EBF means feeding the infant nothing other than breastmilk	Knows	610 (98.2)
	Doesn’t know	11 (1.8)
EBF should last for the first 6 months of life	Knows	610 (98.2)
	Doesn’t know	11 (1.8)
Breastfeeding on demand	Knows	533 (85.8)
	Doesn’t know	88 (14.2)
Benefits of EBF to the baby	Knows	563 (90.7)
	Doesn’t know	58 (9.3)
Benefits of EBF to the mother	Knows	426 (68.6)
	Doesn’t know	195 (31.4)
Ways to keep up breastmilk supply	Knows	559 (90.0)
	Doesn’t know	62 (10.0)
When mother is absent EBF can continue by expressing breastmilk and/or storing	Knows	232 (37.4)
	Doesn’t know	389 (62.6)
Mother should continue EBF if baby is sick	Knows	519 (83.6)
	Doesn’t know	102 (16.4)
Options of breastfeeding for HIV positive mother who chooses to breastfeed	Knows	530 (85.4)
	Doesn’t know	91 (14.6)
Very low birthweight infant should be EBF by skin to skin contact (Kangaroo mother care)	Knows	103 (16.6)
	Doesn’t know	518 (83.4)
A COVID-19 positive/suspect mother should initiate/continue breastfeeding with strict precautions	Knows	220 (35.4)
	Doesn’t know	401 (64.6)

*COVID-19* Corona Virus Disease-19, *EBF* Exclusive Breastfeeding, *HIV* Human Immune Deficiency Virus

### Attitude towards exclusive breastfeeding during COVID-19

Four hundred eighty seven (78.4%) mothers said that they felt good to exclusively breastfeed their babies during COVID-19. Similarly, three-fourth (74.9%) of the mothers said that they felt good to breastfeed their babies on demand during the pandemic. Additionally, three hundred ninety five (63.6%) mothers said that they felt comfortable to follow COVID-19 precautions while breastfeeding. Regarding confidence, 483 (77.8%) mothers said that they were confident to exclusively breastfeed their babies during COVID-19 pandemic (Table 3).

**Table 3:** Attitude towards exclusive breastfeeding during COVID-19 among lactating mothers/caregivers in Mekelle City, Tigrai, 2021, (*n* = 621)

Variable	Frequency	Percentage (%)
Feel good to exclusively breastfeed my baby for 6 months during COVID-19 pandemic	487	78.4
Find it difficult to exclusively breastfeed my baby for 6 months during COVID-19 pandemic	86	13.9
Feel good breastfeeding my baby on demand during COVID-19 pandemic	465	74.9
Find it difficult breastfeeding my baby on demand during COVID-19 pandemic	53	8.5
Feel comfortable to follow COVID-19 precautions while breastfeeding	395	63.6
Find it difficult to follow COVID-19 precautions while breastfeeding	28	4.5
Feel confident to exclusively breastfeed my baby during COVID-19 pandemic	483	77.8
Feel confident to express/store my breast milk to be given to my baby when I am away	147	23.7

*COVID-19* Corona Virus Disease-19

Based on the knowledge and attitude questions, the mean (±SD) knowledge and attitude scores stood at 11.8 (±1.87) out of a maximum 16 and 19.9 (±3.28) out of a maximum 24, respectively (Table 4).

**Table 4:** Summary statistics of knowledge and attitude questions on breastfeeding during COVID-19 pandemic among lactating mothers in Mekelle city, Tigrai, 2021, (*n* = 621)

Variable	Mean (±SD)	Median (IQR)	Minimum	Maximum
Knowledge	11.8 (±1.87)	12 (3)	5	16
Attitude	19.9 (±3.28)	20 (7)	9	24

*IQR* Interquartile range, *SD* Standard deviation

### Practice of exclusive breastfeeding during COVID-19

While all mothers breastfed their babies during the 24 hours preceding the survey, five hundred thirty one (85.5%) mothers said that they fed their babies’ colostrum. Besides, more than half (56%) of the mothers said that they continued breastfeeding when their babies’ were sick. Moreover, 513 (82.6%) and 524 (84.4%) mothers reported breastfeeding their babies’ by proper positioning and proper attachment, respectively.

**Fig. 1:**
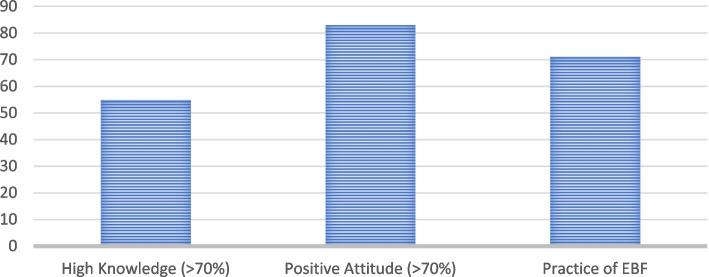
Level of knowledge, attitude, and practice of exclusive breastfeeding during COVID-19 pandemic among lactating mothers/caregivers in Mekelle city, Tigrai, 2021, (*n* = 621)

Among the total 621 mothers, 400 (64.4%) exclusively breastfed their babies. Besides, 341 (54.9%) and 503 (81.0%) mothers had higher knowledge (knowledge score > 70%) and desirable/positive attitude (attitude score > 70%), respectively (Fig. 1).

### Determinants of exclusive breastfeeding during COVID-19

Binary logistic regression analysis was run through two steps to point out significant determinants of EBF during COVID-19 pandemic. Exclusive breastfeeding was the outcome variable with two categorical levels (No = 0, Yes = 1). During the multivariate logistic regression, household head sex, mothers’ educational status, parity, breastfeeding information, knowledge, and attitude were found to be significantly associated with the outcome variable at *p* < 0.05.

Mothers from female-headed households were 2.2 times more likely to exclusively breastfeed their babies (AOR 2.21; 95% CI 1.31, 3.71) compared to those from male headed households. Compared to mothers who didn’t attend formal education, those who attained primary, secondary, and college degree or above education had 1.24 (AOR 1.24; 95% CI 1.10, 1.55), 1.29 (AOR 1.29; 95% CI 1.02, 2.02), and 1.41 times (AOR 1.41; 95% CI 1.22, 2.90) higher odds of EBF, respectively. Additionally, mothers who received breastfeeding related information during their postnatal stay in health facilities had 73% (AOR 1.73; 95% CI 1.17, 2.56) higher odds of EBF than those who did not.

By contrast, mothers who were in the higher knowledge category had about 74% (AOR 1.74; 95% CI 1.20, 2.51) higher likelihood of EBF practice. Likewise, mothers with positive or desired attitude showed a more than twice (AOR 2.35; 95% CI 1.50, 3.70) higher odds of EBF when compared to those with less positive or undesired attitude. An increase in the parity of mothers was associated with a 23% (AOR 1.23; 95% CI 1.07, 1.42) increment in the odds of EBF practice (Table 5).

**Table 5:** Binary logistic regression analysis of determinants of Exclusive breastfeeding during COVID-19 among lactating mothers in Mekelle city, Tigrai, 2021, (*n* = 621)

Variables	Category	EBF		COR (95%CI)	AOR(95%CI)	*p*-value
		No	Yes			
Household head sex	Female	28	93	2.38(1.42,3.98)	2.21(1.31,3.71)	0.003*
	Male (Ref.)	193	307	1	1	–
Mothers’ education	Primary	71	121	1.43 (1.17,1.62)	1.24(1.1,1.55)	0.023*
	Secondary to preparatory	93	144	1.49 (1.22,2.33)	1.29(1.02,2.02)	0.009*
	College or above	47	128	1.82(1.72,3.10)	1.41(1.22,2.90)	0.005*
	No formal education (Ref.)	10	7	1	1	–
Child age	Mean (SD)	2.96(1.40)	2.78(1.39)	0.87(0.77,0.98)	0.90(0.79,1.02)	0.11
Parity	Mean (SD)	2.42(1.40)	2.70(1.56)	1.16(1.03,1.32)	1.23(1.07,1.42)	0.004*
ANC	Yes	207	395	5.34(1.89,15.04)	2.99(0.96,9.30)	0.06
	No (Ref.)	14	5	1	1	–
Breastfeeding information received during postnatal care	Yes	140	306	1.88(1.32,2.70)	1.73(1.17,2.56)	0.006*
	No (Ref.)	81	94	1	1	–
Maternal occupation	Employed	58	162	1.91(1.33,2.74)	1.30(0.84,2.04)	0.24
	Housewife (Ref.)	163	238	1	1	–
Knowledge	High (> 70%)	91	250	2.38(1.70,3.33)	1.74(1.20,2.51)	0.003*
	Low (<=70%) (Ref.)	130	150	1	1	–
Attitude	Desired/positive	154	349	2.98(1.97,4.49)	2.35(1.50,3.70)	< 0.001**
	Less positive (Ref.)	67	51	1	1	–

*ANC* Antenatal Care, *AOR* Adjusted Odds Ratio, *COR* Crude Odds Ratio, *COVID-19* Corona Virus Disease-19, *CI* Confidence Interval, *EBF* Exclusive Breastfeeding, *Ref* reference

* = *p* < 0.05; ** = *p* < 0.001

## Discussion

This study was conducted to assess the KAP of lactating mothers towards EBF and its determinants during the COVID-19 pandemic. The response rate was 98.1% with 621 out of the total 633 mothers having completed the study.

### Gaps in knowledge and attitude of EBF during COVID-19

This study found out that slightly greater than half of the sampled mothers had a higher knowledge score (score > 70%) with a mean knowledge score of 11.8 ± 1.87 out of a maximum 16. Besides, about two-third of the mothers did not know that a COVID-19 positive/suspect lactating mother should initiate/continue breastfeeding under strict precautions. Similarly, studies done in Ethiopia [19], Ghana [20], and Nigeria [21] before the pandemic reported inadequate knowledge related to EBF. A study done in Turkey during the COVID-19 pandemic also showed similar figs [22].. During the pandemic, mothers are supposed to start/keep breastfeeding as long as adequate precautions to avoid mother-infant transmission are followed [7, 23].

Even symptomatic COVID-19 positive lactating mothers could feed their infants by expressing breastmilk [9]. This could be because horizontal transmission from the mother to the infant through breastmilk is unlikely [10, 24]. As the host of benefits of EBF and skin to skin contact outweigh the risks of transmission, separation of the mother from her baby might not be a good option [25]. For more conclusive and data-driven recommendations, however, pregnant and lactating women should be included in COVID-19 clinical trials [26].

Regarding attitude, majority of the mothers in our study were positively inclined towards EBF during COVID-19 pandemic. Others studies also showed similar findings [19, 27]. In Australia, mothers who had not initiated or had stopped to breastfeed due to the COVID-19 pandemic were highly motivated and sought support from Australian breastfeeding association to initiate/restart breastfeeding [28]. Motivational interviewing has been recognized as one successful technique for applying tailored breastfeeding counseling and educational programs by decreasing uncertainty and increasing attitude towards EBF [29, 30].

### Exclusive breastfeeding practice and its determinants

Among the total surveyed lactating mothers, about two-third (64.4%) practiced EBF. Although this proportion is not very low, it can never be considered a success, especially considering the study area, where urban people have better healthcare, media, and other services exposure. Findings from other studies are inconclusive. While some studies [31, 32] found similar practice levels, other studies [15, 33] showed higher practice levels, and some others [34–36] reported far lower levels compared to our findings. Such inconsistencies in the findings could be attributed to various factors including differences in study area, sample size, study period, and other social and health service related factors.

A recent study has shown that significant number of mothers who tested positive for COVID-19 continued to breastfeed up to 6 months postpartum [37]. Mothers should always be encouraged to breastfeed. Even in trying times like the COVID-19 pandemic, breastfeeding creates a bonding between the mother and the child that in turn carries a positive vice-versa effect in promoting good breastfeeding practices and eventually optimal nutrition.

Our study found out that mothers from female-headed households were more likely to practice EBF in contrast to their counterparts. When mothers are the heads of their households, it means that they have the upper hand in making decisions regarding household and family matters, among others, including child feeding practices.

Compared to those who didn’t attend formal education, mothers who attended primary, secondary to preparatory, and college or above education showed higher odds of EBF practice. Elsewhere, primary or higher educational status of mothers was associated with higher odds of breastfeeding up to 6 months of age [32, 38]. Mothers with higher educational status might be very likely to have greater exposure to breastfeeding-related information and to translate such information into practice.

Moreover, in the present study, an increase in parity was associated with increased odds of EBF practice. This association was supported by a study done in Kenya which showed a 39.4% rate of EBF among primiparous mothers compared to a 49.3% prevalence among multiparous ones [39]. In this sense, it is necessary to provide primiparous with more breastfeeding related information during pre or/and postnatal periods. Mothers with more frequent birth experiences would obviously be occupied with better knowledge and thus intention and practice of EBF.

Additionally, our study showed that mothers who received breastfeeding information during their postnatal stay had 73% higher odds of EBF than those who didn’t. A study conducted in Nigeria has underlined the essentiality of disseminating information about EBF by various health care providers and agencies to mothers and their families in improving child feeding and nutrition [40]. However, healthcare providers and policymakers should also consider the likelihood that COVID-19, along with its lockdown measures, may cause depression and anxiety during early postpartum period and thus interfering with breastfeeding [41].

By contrast, mothers who were in the higher knowledge category had about 74% higher likelihood of EBF practice. Providing tailored counseling and awareness creating sessions to lactating mothers and people around them could increase their knowledge and then practice of EBF [42]. Several international or/and regional organizations have emphasized the need for improving the knowledge of lactating mothers regarding breastfeeding by describing its short and long-term immunologic, nutritional, and health benefits for both the mother and child. Responsible stakeholders also need to put a great deal of concerted efforts to properly advise and support mothers with breastfeeding, even during moments when the mothers have confirmed or suspected COVID-19 infection [10].

In this study, mothers with positive or desired attitude showed more than twice higher odds of EBF when compared to those with less positive or undesired attitude. Quite logically, mothers positively inclined towards breastfeeding are more likely to continue breastfeeding and have a greater chance of successful EBF practice. Hence, evidence supports that antenatal and early postpartum education and counseling related to breastfeeding could improve maternal attitudes and knowledge toward optimal breastfeeding practices [43, 44].

### Strengths and limitations

One strength of this study is that it was the first study in Ethiopia to assess lactating mothers’ knowledge, attitude, and determinants of EBF during the COVID-19 pandemic. Second, generalizability of the findings could be assured as it used a fairly large sample size. However, this study had some limitations. First, although the data collection process went smoothly, the breaking out of war in Tigrai might had, in some way, interfered in the process of conducting the study. Second, even though we tried to control for the effect of possible confounders at all levels of the study, their effect can never be ruled out.

## Conclusions

To sum up, nearly two-thirds of the mothers practiced EBF. Despite the recommendations provided by WHO and other organizations on the benefits of EBF during the COVID-19 pandemic, our findings indicated inadequate knowledge, attitude, and practice of EBF.

Household head, maternal educational status, parity, breastfeeding information, knowledge of breastfeeding, and attitude towards breastfeeding were significant determinants of EBF. Hence, this study implied that developing programs that enhance women’s participation in education and decision-making could improve EBF practice. Besides, providing lactating mothers with adequate and up-to-date breastfeeding information during the COVID-19 pandemic could be significant in improving breastfeeding practices.

## Data Availability

Data supporting the findings in this paper are available upon reasonable request from the corresponding author and the summary data are available in the main document.

## Abbreviations

ANC: Antenatal Care
AOR: Adjusted Odd Ratio
CI: Confidence Interval
COVID-19: Corona Virus Disease-2019
COR: Crude Odd Ratio
EBF: Exclusive Breast Feeding
IQR: Inter-Quartile Range
KAP: Knowledge, Attitude, and Practice
SD: Standard Deviation
WHO: World Health Organization
